# Epidemiology of rotavirus diarrhea among children less than 5 years hospitalized with acute gastroenteritis prior to rotavirus vaccine introduction in India

**DOI:** 10.1016/j.vaccine.2020.10.084

**Published:** 2020-12-03

**Authors:** C.P. Girish Kumar, Sidhartha Giri, Mamta Chawla-Sarkar, Varanasi Gopalkrishna, Shobha D. Chitambar, Pratima Ray, S. Venkatasubramanian, Biswajyoti Borkakoty, Subarna Roy, Jyothi Bhat, Bhagirathi Dwibedi, Vijayachari Paluru, Pradeep Das, Rashmi Arora, Gagandeep Kang, Sanjay M. Mehendale, National Rotavirus Surveillance Network investigators

**Affiliations:** a ICMR-National Institute of Epidemiology, Chennai, Tamil Nadu, India; b Division of Gastrointestinal Sciences, Christian Medical College, Vellore, Tamil Nadu, India; c ICMR-National Institute of Cholera and Enteric Diseases, Kolkata, West Bengal, India; d ICMR-National Institute of Virology, Pune, Maharashtra, India; e Jamia Hamdard, New Delhi, India; f ICMR-Regional Medical Research Centre, Dibrugarh, Assam, India; g ICMR-National Institute of Traditional Medicine, Belgaum, Karnataka, India; h ICMR-National Institute for Research in Tribal Health, Jabalpur, Madhya Pradesh, India; i ICMR-Regional Medical Research Centre, Bhubaneswar, Odisha, India; j ICMR-Regional Medical Research Centre, Port Blair, Andaman & Nicobar Islands, India; k ICMR-Rajendra Memorial Research Institute of Medical Sciences, Patna, Bihar, India; l Indian Council of Medical Research (ICMR), New Delhi, India

**Keywords:** Rotavirus, Surveillance, Diarrhea, Children, India

## Abstract

**Background:**

Rotavirus is an important cause of severe diarrhea requiring hospitalization, accounting for approximately 78,000 deaths annually in Indian children below 5 years of age. We present epidemiological data on severe rotavirus disease collected during hospital-based surveillance in India before the introduction of the oral rotavirus vaccine into the national immunization schedule.

**Methods:**

The National Rotavirus Surveillance Network was created involving 28 hospital sites and 11 laboratories across the four geographical regions of India. From September 2012 to August 2016 children less than 5 years of age hospitalized for diarrhea for at least 6 h, were enrolled. After recording clinical details, a stool sample was collected from each enrolled child, which was tested for rotavirus antigen using enzyme immunoassay (EIA). Nearly 2/3rd of EIA positive samples were genotyped using reverse transcription polymerase chain reaction to identify the G and P types.

**Results:**

Of the 21,421 children enrolled during the 4 years surveillance, 36.3% were positive for rotavirus. The eastern region had the highest proportion of rotavirus associated diarrhea (39.8%), while the southern region had the lowest (33.8%). Rotavirus detection rates were the highest in children aged 6–23 months (41.8%), and 24.7% in children aged < 6 months. Although rotavirus associated diarrhea was seen throughout the year, the highest positivity was documented between December and February across all the regions. The most common rotavirus genotype was G1P[8] (52.9%), followed by G9P4 (8.7%) and G2P4 (8.4%).

**Conclusions:**

There is high burden of rotavirus gastroenteritis among Indian children below 5 years of age hospitalized for acute diarrhea thereby highlighting the need for introduction of rotavirus vaccine into the national immunization program and also for monitoring circulating genotypes.

## 1 Introduction

Globally, rotavirus is the most frequent cause of severe acute gastroenteritis (AGE)/acute watery diarrhea among young children. Although viral gastroenteritis is generally self-limiting, rotavirus can cause severe, dehydrating diarrhea resulting in an estimated two million hospitalizations and over 25 million outpatient visits globally [1]. About 215,000 children died worldwide in 2013 from rotavirus associated diarrhea, and the majority of these deaths occurred in low-income countries [2]. Revised estimates from India from 2011 to 2013 have reported that approximately 78,000 children die due to rotavirus gastroenteritis annually, with about 59,000 in the under 2 years age group [3].

Effectiveness demonstrated by both the internationally licensed rotavirus vaccines, Rotarix® (GlaxoSmithKline Biologicals) and Rotateq® (Merck & Co) in introducer countries, supported the World Health Organization (WHO) in recommending the inclusion of rotavirus vaccine in the national immunization programs of countries especially in Asia and sub-Saharan Africa, where the burden of rotavirus disease is high. WHO has reiterated the use of rotavirus vaccination as part of a comprehensive strategy to control diarrheal diseases with scaling up of preventive strategies such as proper handwashing, improved water, and sanitation as well as treatment modalities including low osmolarity oral rehydration solution (ORS) and zinc [2]. Since 2015, two indigenously developed oral rotavirus vaccines (ROTAVAC® by Bharat Biotech; ROTASIIL® by Serum Institute of India) have been licensed by the Drug Controller General of India (DCGI), and both obtained the WHO prequalification in 2018 [4].

For considering the introduction of rotavirus vaccine in the national immunization program in India, robust data on rotavirus burden was needed. To address this data gap, a nation-wide sentinel surveillance platform called “National Rotavirus Surveillance Network (NRSN)” was established in 2005 by Indian Council of Medical Research- the apex body responsible for conducting medical research in India. NRSN was expanded in 2012 for generating more recent and nationally representative data on rotavirus disease burden. Details on setting up of the NRSN and the initial findings from the surveillance has been previously reported [5], [6], [7], [8].

The purpose of the present study was to generate basic epidemiological data that could contribute to evidence-based decision making by the Government of India on phased introduction of the rotavirus vaccine (ROTAVAC, ROTASIIL) in India from 2016. In this paper, we report the epidemiological profile in terms of rotavirus burden in India (2012–2016) by host characteristics like age, gender, severity of illness as well as regional, seasonal or genotypic variations in the circulating virus during the surveillance period.

## 2 Materials and methods

### 2.1 Study setting

Hospital-based surveillance was carried out among children under 5 years of age hospitalized for management of acute watery diarrhea at 28 pediatric hospitals or medical college hospitals covering 19 states/union territories in India [7]. Surveillance was carried out between September 2012 and August 2016. Coordination of surveillance activities across the network and data management was carried out by the ICMR - National Institute of Epidemiology in Chennai, India. The Epidemiology and Communicable Diseases (ECD) Division of ICMR was responsible for overall administrative and technical coordination of the surveillance network.

### 2.2 Ethics

The study was approved by the institutional ethics committees of all the participating centers/sites. Written informed consent was obtained from the parent or guardian before each child was enrolled.

### 2.3 Case enrolment and clinical assessment

Details of case ascertainment and enrolment in NRSN has been previously described [6]. Briefly, all cases of acute watery diarrhea among children aged (0–59 months) admitted to the participating hospital for supervised oral or intravenous rehydration were considered for enrolment. Demographic and clinical details were recorded on a structured case reporting form. Diarrheal disease severity was determined using the modified Vesikari severity scoring system (≤5 mild; 6–10 moderate; 11–15 severe, and 16–20 very severe).

### 2.4 Sample collection and testing

Stool specimens were collected and tested for the presence of group A rotavirus antigen using the enzyme immunoassay (EIA) (Premier® Rotaclone® Meridian Bioscience). Rotavirus positive stool specimens (20% W/V) were subjected to RNA extraction using Qiagen viral RNA kit, followed by reverse transcription and hemi-nested multiplex polymerase chain reaction (PCR) for amplification of the VP7 and VP4 regions to identify G and P types, respectively [9], [10]. Genotyping was initially performed on every rotavirus positive stool specimen, and thereafter systematic testing of every third positive sample was carried out. Quality assurance of the laboratory component involved annual proficiency testing by participating laboratories and concordance testing of 10% of study specimens at the surveillance network’s coordinating laboratory at Christian Medical College (CMC), Vellore, India.

### 2.5 Data analysis

Data were analyzed to assess the proportions (unadjusted) of rotavirus-positive cases with respect to demographic factors, symptoms, disease severity, median duration of hospitalization, genotype distribution, and also by season and regions. Variable categories viz. age, season, and region showing significance in chi-square test were further subjected to pair-wise comparison by post hoc chi-square test with Bonferroni correction. Analyses were carried out using SPSS v. 20.0 and Stata v.10.0.

## 3 Results

**Fig. 1:**
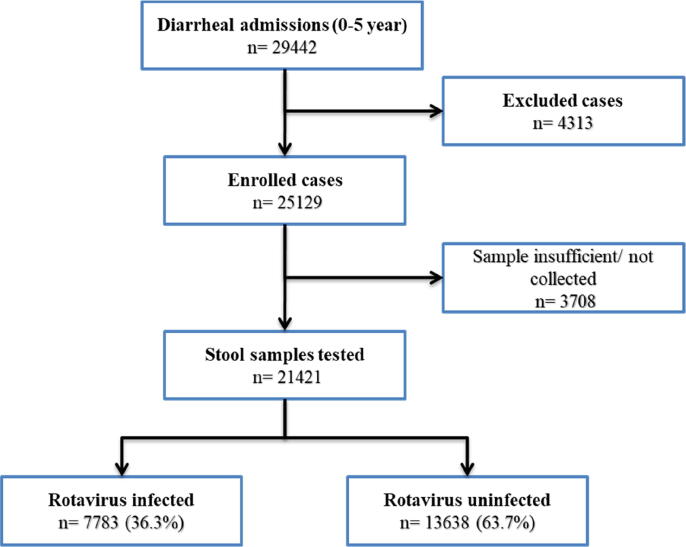
Flow diagram summarizing case enrolment and rotavirus detection.

Between September 2012 and August 2016, 25,129 children presenting with acute watery diarrhea requiring hospitalization were enrolled in the surveillance at participating surveillance hospitals. Stool samples were collected from >85% of enrolled children, whereas in the remaining enrollees, stool was either not collected or the quantity of sample collected was insufficient for testing. Among the 21,421 stool samples tested (Fig. 1), rotavirus antigen was detected in 7783 (36.3%) samples (Table 1). There was a significant difference in the proportion of rotavirus gastroenteritis across the four geographical regions (p < 0.001) (Table 2). The eastern region had the highest proportion of rotavirus associated diarrhea (39.8%), followed by north (37.7%). The lowest burden of rotavirus infection was seen in the southern region (33.8%). Site wise, rotavirus positivity was the highest in Bhubaneswar in Odisha state of India (54.9%), followed by Midnapore (West Bengal; 53.5%), whereas the lowest positivity rates were seen in Nalanda (14.5%) and Patna (17.5%) sites in Bihar (Table 1).

**Table 1:** Site-wise distribution of rotavirus positivity in India [2012 – 2016].

Region	State	CRS	Children Admitted	Children Enrolled	Samples Tested	RV Positive	%
**East**	**West Bengal**	Kolkata	1754	1457	1367	680	49.7
		Midnapore	1830	1171	1063	569	53.5
	**Assam**	Dibrugarh	693	680	529	203	38.4
	**Nagaland**	Dimapur	614	614	546	220	40.3
	**Bihar**	Patna	1172	953	967	169	17.5
		Nalanda	762	748	662	96	14.5
	**Odisha**	Bhubaneswar	2020	1281	712	391	54.9
	Total		**8845**	**6904**	**5846**	**2328**	**39.8**
**West**	**Maharashtra**	Pune	1179	1058	804	389	48.4
		Mumbai	771	706	629	189	30.0
		Karad	1014	1000	844	294	34.8
	**Gujarat**	Ahmedabad	512	510	400	92	23.0
		Surat	1249	1207	1033	308	29.8
	Total		**4725**	**4481**	**3710**	**1272**	**34.3**
**South**	**T N**	Vellore	1760	1549	1337	392	29.3
		Trichy	679	655	561	277	49.4
		Chennai	1493	1336	1123	338	30.1
	**A N**	Port Blair	1557	1557	1453	593	40.8
	**Kerala**	Kolenchery	1282	1143	899	389	43.3
	**Telangana**	Hyderabad	1056	686	663	164	24.7
	**Andra Pradesh**	Tirupati	826	798	784	184	23.5
	**Karanataka**	Belgaum	625	615	574	162	28.2
	Total		**9278**	**8339**	**7394**	**2499**	**33.8**
**North**	**Punjab**	Ludhiana	751	748	569	213	37.4
	**Himachal Pradesh**	Tanda	710	513	454	241	53.1
	**Uttar Pradesh**	Meerut	528	515	458	83	18.1
	**New Delhi**	Delhi*	2666	2105	1864	844	45.3
	**Haryana**	Rohtak	1040	655	538	161	29.9
	**Madhya Pradesh**	Jabalpur	505	475	336	94	28.0
		Bhopal	394	394	252	48	19.0
	Total		**6594**	**5405**	**4471**	**1684**	**37.7**
	**India**		**29,442**	**25,129**	**21,421**	**7783**	**36.3**

* Comprised two clinical recruitment sites.

**Table 2:** Characteristics of rotavirus infected and uninfected children.

Variable		Rotavirus infected children [n (%)] (N = 7783)	Rotavirus un-infected children [n (%)] (N = 13638)	p value
Age (months)	0–2	253 (17.0)	1235 (83.0)	<0.001
	3–5	700 (29.4)	1678 (70.6)	
	6–11	2743 (40.7)	3998 (59.3)	
	12 to 23	2862 (43.1)	3782 (56.9)	
	24 to 35	734 (33.0)	1488 (67.0)	
	≥36	491 (25.2)	1457 (74.8)	
Sex	Male	4839 (36.5)	8423 (63.5)	0.550
	Female	2944 (36.1)	5215 (63.9)	
Disease severity	Mild–Moderate (VS 1–10)	2785 (35.8)	5944 (43.6)	<0.001
	Severe to Very Severe (VS 11–20)	4998 (64.2)	7694 (56.4)	
Rotavirus vaccination	Yes	223 (30.4)	511 (69.6)	0.001
	No	7560 (36.5)	13,127 (63.4)	
Region	East	2328 (39.8)	3518 (60.2)	<0.001
	West	1272 (34.3)	2438 (65.7)	
	South	2499 (33.8)	4895 (66.2)	
	North	1684 (37.7)	2787 (62.3)	
Season	Dec–Feb	3302 (53.2)	2910 (46.8)	<0.001
	Mar–May	1721 (33.2)	3455 (66.8)	
	Jun–Aug	1097 (20.2)	4336 (79.8)	
	Sep–Nov	1663 (36.2)	2937 (63.8)	

VS-Vesikari score.

About two-thirds of the enrolees were male children. However, there was no significant difference in the gender-wise proportion of rotavirus positivity [male (36.5%) versus female (36.1%); p = 0.5509] (Table 2). Over 80% of children enrolled in the surveillance were under two years of age, and the burden of rotavirus associated diarrhea was highest in this age group (38%). The proportion of rotavirus positive cases in children less than three months old was 17%, whereas it was highest among children aged 12–23 months (43.1%) (Table 2). There was a decrease in positivity with an increase in age beyond 2 years. Median age (interquartile range; IQR) for rotavirus positive cases was 12 months (8–18 months), whereas for rotavirus negative cases was 11 months (6–20 months), and this difference was statistically significant (p = 0.000).

**Fig. 2:**
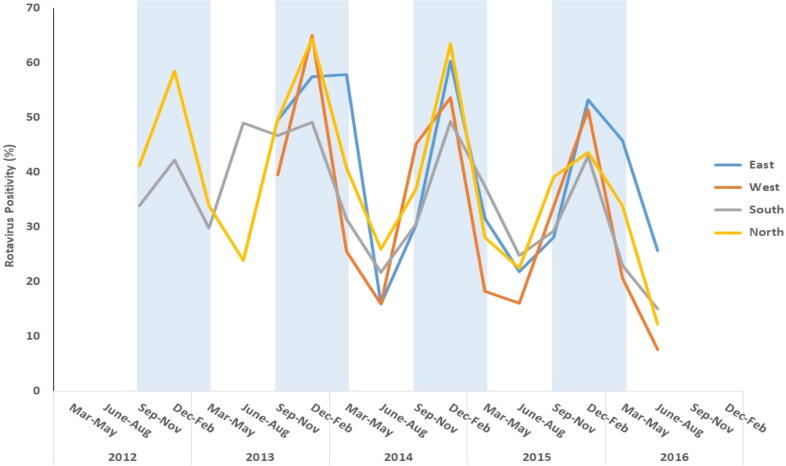
Seasonal pattern of rotavirus infection across different regions of India. [Vertical blue bars represent rotavirus peak seasons] (For interpretation of the references to colour in this figure legend, the reader is referred to the web version of this article.)

Rotavirus associated diarrhea was reported throughout the year with the highest positivity seen during December- February (53.2%) followed by September – November (36.2%) (Fig. 2). The rates of rotavirus detection were generally lower during the non-peak times of June –August and particularly in 2016, when only 8%–15% positivity was seen in west, north, and south zones. Post hoc chi-square analysis showed that with the exception of 3–5 months vs. 24–35 months and 6–11 months vs. 12–23 months, the differences in incidence of rotavirus infection in various pairs of age groups were statistically significant (p < 0.05). Similarly, pair-wise comparison of regions except for East vs. North and West vs. South showed a statistically significant difference. Analysis of seasons showed that pair-wise differences in all categories were statistically significant.

About 60% of all hospitalized children with AGE had severe to very severe diarrhea (Vesikari score > 11). Among rotavirus infected children, 64.2% cases had severe to very severe diarrhea, and this was significantly higher than the proportion of severe to very severe diarrhea among rotavirus negative cases (p < 0.001; Table 2.). Six or more diarrheal episodes were reported by 81.3% of cases, and the proportion of such cases was significantly higher among rotavirus infected children (p < 0.01). Similarly, there was a significantly higher proportion of rotavirus positive cases who reported vomiting (p < 0.01).

Of the total enrolled, 82.4% of children were managed with intravenous (IV) fluid rehydration. The proportion of children who received IV fluids was similar among rotavirus infected and uninfected groups. IV fluid usage was highest in the eastern region (98.4%), followed by the western region (94.6%), whereas it was 77.5% in the south. Only in about half of the children in the northern region, IV fluids were administered. Only a small proportion (3.5%, 734/21421) of study participants had a history of rotavirus vaccination. The difference of rotavirus diarrhea among the vaccinated children (30.4%, 223/734) and unvaccinated children (36.5%, 7560/20687) was statistically significant (p = 0.001). Over 90% of children with rotavirus vaccine exposure were seen in private hospitals.

Thirty-nine (0.18%) of the enrolled children failed to recover. More than half of the deaths (56.4%) were from the northern region, and these included all nine rotavirus positive cases. The median age of these cases was 5 months (IQR 1.5–12 months), and 72% were <12 months of age. On admission, these cases had a median Vesikari score of 10 (IQR 9–14), and their median length of hospital stay was 4 days (IQR 1–10 days). Nineteen of these children had severe to very severe diarrhea with a median Vesikari score of 14 (IQR 14–15) and a median length of hospital stay of 5 days (IQR 1–15 days). Analysis of the cause of death revealed that mortality in most of the children (n = 36) was attributable to sepsis, shock, seizures, or meningitis associated with severe acute gastroenteritis, whereas in the remaining 3 cases, there was associated bronchopneumonia, liver disease, and milk-aspiration.

Of the 4041 rotavirus positive stools that were subjected to G/P typing, 93% (3757/4041) were genotyped, 4.70% (190/4041) were partially typed, and 2.33% (94/4041) could not be typed. G1P[8] was the commonest genotype (52.9%; 2139/4041), followed by G9P[4] (8.74%; 353/4041) and G2P[4] (8.44%; 341/4041).

## 4 Discussion

This study provides comprehensive data on the burden of rotavirus associated diarrhea among children under five years of age in India. Rotavirus accounted for about 36% of diarrheal admissions among under-five children admitted in 28 hospitals across India during the period of surveillance. This finding is consistent with the findings from earlier multi-centric surveillance conducted between 2005 and 2009 as well as other studies from India that have all documented substantial burden of rotavirus associated diarrhea in the country [11], [12], [13], [14], [15], [16], [17], [18], [19]. A recent global review has reported that the proportion of rotavirus gastroenteritis increases with the increasing severity, ranging from 8 to 10% of diarrheal episodes of all severity to nearly 35–40% of diarrheal episodes requiring hospitalization [20].

Our surveillance detected rotavirus all-round the year, with the annual burden ranging between 33% and 44% across the years and with most of the cases seen during the cooler months of November through February. An increased incidence of rotavirus infections during winter or cooler months of the year have been well documented from several parts of the globe [1], [21], [22]. Because the survival of rotavirus is better in cooler conditions with low relative humidity, it has been hypothesized that a relative drop in humidity and rainfall combined with drying of soils might increase the aerial transport of dried, contaminated fecal material containing rotavirus [21], [22]. Findings from previous Indian studies have also corroborated this [5], [13], [15], [17], [18], [19].

Among the diarrheal hospitalizations, male children were more frequent, but there was no significant difference in rotavirus positivity between the two genders. Male children getting preferential care is a common societal practice in many Indian communities, and the male preponderance among acute diarrheal hospitalizations could be a reflection of this gender-related differential access to health care [23]. It is quite possible that several girls suffering from rotaviral diarrhea might not even get treated and meet fatal outcomes.

Features of clinical presentation, such as the number of diarrheal episodes (≥6), vomiting, and disease severity, were significantly associated with acute rotavirus diarrhea. This finding is similar to data from previous studies conducted in India and elsewhere, which have documented rotavirus associated diarrheal admissions presenting with severe disease [5], [13], [18], [19], [24], [25], [26], [27].

Rotavirus infections are reportedly highest among children under two years of age, and the age distribution of rotavirus positive children in the present surveillance was also consistent with the previous reports [5], [13], [15], [18], [19].

Diversity of rotavirus strains was seen across the country with G1P[8], G9P[4], and G2P[4] as the commonly detected strains. However, the emergence and increasing trend of strains such as G3P[8] were observed towards the end of the surveillance period (data not shown). It will be necessary to continue monitoring the rotavirus strains to assess the impact of rotavirus vaccination on strain distribution and diversity.

Our four years’ study demonstrates the early age-related incidence of rotavirus gastroenteritis in India. The proportion of rotavirus associated diarrhea requiring hospitalization was 17% in children below 2 months of age and 29% in children aged 3–5 months. Similar findings have been reported in earlier surveillance studies from India [5], [13], [19]. The comparatively lower proportion of rotavirus infection seen among children in early infancy (<2 months) could probably be due to the presence of maternal antibodies that passively protect children from severe diarrhea; with the possible waning of maternal antibody levels, children beyond six months more commonly present with severe diarrhea following rotavirus infection [17]. Based on these results, it seems important to focus on younger Indian children for vaccine induced protective immunity. Implications of these findings on the present national policy of rotavirus vaccination programme need to be discussed. However, the majority of rotavirus diarrhea associated admissions were seen in children aged 6–23 months (41.9%). Repeated infections are known to confer protection from severe disease, and this was evidenced by the observed lower rotavirus positivity among children above two years of age in our study [28].

In about two-thirds of the children, diarrhea was not associated with rotavirus, and since the focus of the surveillance was limited to rotavirus, data on the other diarrheal etiologies in these children was not generated. However, studies such as the Global Enteric Multicentre Study (GEMS) and Malnutrition and Enteric Disease study (MAL-ED) have provided valuable insights on the major etiologies of diarrheal disease among young children in study sites from several countries, including India [29], [30]. In the multi-centric case-control GEMS study involving four study sites in Africa and three in Asia, the most diarrheal cases during 2007–2011 were attributable to rotavirus, *Cryptosporidium*, *Shigella*, and enterotoxigenic *Escherichia coli* (ETEC). Kolkata, a surveillance site from India in the GEMS study, had an attributable rotavirus fraction of moderate to severe diarrhea cases as 27% in 0–11 month and 25.4% in 12–23 month age groups [29]. In the present surveillance, Kolkata has consistently documented a high burden of rotavirus associated AGE.

In the MAL-ED birth cohort study conducted between 2009 and 2014 across eight sites in Asia, Africa, and South America, the most common agents associated with diarrhea were norovirus GII, rotavirus, *Campylobacter* spp., astrovirus, and *Cryptosporidium* during infancy and *Campylobacter* spp, norovirus GII, rotavirus, astrovirus, and *Shigella* during the second year of life. In Vellore, which was the site from India in the MAL-ED study, rotavirus was the predominant etiology causing diarrhea in the first 2 years of life with an attributable fraction of 6% in 0–11 month and 4.8% in 12–23 month age groups [30]. In the current surveillance, Vellore showed a higher proportion of rotavirus associated diarrhea in hospitalized children.

In the Global Burden of Disease 2015 study that assessed diarrheal mortality in 195 countries and territories from 1990 to 2015, rotavirus was the leading pathogen associated with mortality among children younger than 5 years in 2015, causing an estimated 146,000 deaths (95% UI 118000–183000), followed by *Cryptosporidium* spp (60400 deaths, 13709.1–134506.4), and *Shigella* spp (54900 deaths, 27000–94700) [31]. These three etiologies accounted for >50% of deaths due to diarrhea in this age group.

Both GEMS and MAL-ED studies emphasized the public health importance of rotavirus in countries where rotavirus vaccination was not yet introduced. In the MAL-ED study, rotavirus had the highest attributable fraction (AF) for sites without vaccine introduction, whereas in sites with vaccine introduction, it had the fifth-highest AF. The decline in rotavirus diarrhea has been well documented in several rotavirus vaccine introducer countries. With the introduction of the rotavirus vaccine in India, it will be necessary to continue monitoring trends of not only rotavirus associated diarrhea but also the other diarrheal etiologies. Such data will aid health authorities in strategizing for appropriate interventions for prevention and/or control of pediatric diarrheas, which are not associated with rotavirus.

As of April 2020, 107 countries have introduced rotavirus vaccines in their national immunization programs [32]. Globally, the introduction of the Rotarix and Rotateq vaccines was followed by significant declines in the proportion of rotavirus associated acute gastroenteritis cases among children below 5 years of age. Data from multiple countries and regions have demonstrated the effectiveness of both these vaccines in low, medium, and high mortality settings. The vaccine efficacy of newer WHO prequalified vaccines, ROTAVAC (G9P[11]) and ROTASIIL (G1-4, G9), has been shown under phase III trial conditions against disease burden as well as major rotavirus genotypes that included the genotypes commonly observed in the present surveillance. ROTAVAC demonstrated vaccine efficacy of 55.1% in children up to two years of age [33]. Vaccine efficacy of ROTAVAC in the second year of life was slightly less than that in the first year of life (48.9% vs. 56.3%). Primary analysis efficacy for ROTASIIL against severe and very severe rotavirus gastroenteritis was 39.5% and 60.5%, respectively [34]. With higher rotavirus disease incidence in low and middle-income countries such as India, even with lower efficacy, a substantial reduction in severe gastroenteritis and death is anticipated following vaccine rollout.

There were a few limitations to our study. These include - i) Surveillance hospitals were mostly tertiary care facilities, and therefore the profile of cases attending primary or secondary care centers have not been adequately captured. ii) Lack of proper awareness of diarrheal management with oral rehydration coupled with limited or poor access to health care, often leads to diarrhea associated deaths among young children in the community. In contrast, most of the children reaching hospitals have better outcomes. This may have resulted in a fewer number of deaths documented during the surveillance period. iii) Data on bacterial and other viral etiologies of pediatric diarrhea was not available from this surveillance activity. Such data would have been useful to monitor trends in the burden of diarrhea attributable to these agents in the post-rotavirus vaccine introduction scenario in India.

## 5 Conclusion

With the introduction of the rotavirus vaccine into the national immunization programme, it will be important to monitor the rates of rotavirus gastroenteritis in the non-vaccinated children in the older age groups because they will be reflective of herd immunity, which is a cumulative benefit of expanding rotavirus vaccination. With the bulk of rotavirus infections seen in children <2 years of age, administration of rotavirus vaccine to age-eligible children has the potential to substantially reduce the burden of both morbidity and mortality associated with rotavirus diarrhea. It will also be important to continuously monitor the prevalent genotypes because they could provide critical inputs in vaccine design in the years to come. Continued surveillance for rotavirus infection after the introduction of the rotavirus vaccine will be critical to assess the impact of the vaccine on rotavirus associated hospitalization rates, geographic spread, and mortality in Indian children suffering from diarrhea at early age.

## Declaration of Competing Interest

The authors declare that they have no known competing financial interests or personal relationships that could have appeared to influence the work reported in this paper.
